# K^+^ channels and cell cycle progression in tumor cells

**DOI:** 10.3389/fphys.2013.00220

**Published:** 2013-08-20

**Authors:** Halima Ouadid-Ahidouch, Ahmed Ahidouch

**Affiliations:** ^1^Laboratory of Cellular and Molecular Physiology EA4667, SFR CAP-SANTE FED 4231, UFR Sciences, University of Picardie Jules VerneAmiens, France; ^2^Department of Biology, Faculty of Sciences, Ibn Zohr UniversityAgadir, Morocco

**Keywords:** K^+^ channels, cell cycle, tumor cell, cyclins, CDK, membrane potential, calcium, volume control

## Abstract

K^+^ ions play a major role in many cellular processes. The deregulation of K^+^ signaling is associated with a variety of diseases such as hypertension, atherosclerosis, or diabetes. K^+^ ions are important for setting the membrane potential, the driving force for Ca^2+^ influx, and regulate volume of growing cells. Moreover, it is increasingly recognized that K^+^ channels control cell proliferation through a novel signaling mechanisms triggered and modulated independently of ion fluxes. In cancer, aberrant expression, regulation and/or sublocalization of K^+^ channels can alter the downstream signals that converge on the cell cycle machinery. Various K^+^ channels are involved in cell cycle progression and are needed only at particular stages of the cell cycle. Consistent with this idea, the expression of Eag1 and HERG channels fluctuate along the cell cycle. Despite of acquired knowledge, our understanding of K^+^ channels functioning in cancer cells requires further studies. These include identifying the molecular mechanisms controlling the cell cycle machinery. By understanding how K^+^ channels regulate cell cycle progression in cancer cells, we will gain insights into how cancer cells subvert the need for K^+^ signal and its downstream targets to proliferate.

## Introduction

Cell homeostasis requires a delicate balance between formation of new cells by cell proliferation and their elimination by apoptosis. Cell proliferation involves, at some point, activation of Cl^−^, K^+^, aquaporin, and Ca^2+^ channels (Pardo, 2004; Lang et al., 2007; Lehen'kyi et al., 2007). As the respective channel inhibitors have been reported to interfere with cell proliferation, the channels appear to play an active role in the machinery triggering cell entry and progression through the cell cycle (Wonderlin and Strobl, 1996; Ouadid-Ahidouch and Ahidouch, 2008; Becchetti, 2011).

Perturbations in the cell cycle results in unlimited proliferation and confers the apoptosis resistance leading to cancer development (Malumbres and Barbacid, 2001, 2009). The progression of cells through the cell cycle is regulated by different cyclin/Cyclin-Dependent Kinase (CDKs) complexes. The cyclin-CDK complexes govern a linear progression of the events that lead cells from a resting state (G0), to growth phase (G1), DNA replication phase (S), and finally to cell division (M). Cyclins D (D1, D2, and D3) and their associated CDKs (4/6) are important during the G1 phase (Caldon et al., 2006). Cyclins E and A, along with their partner, CDK2, are important for the G1/S phase transition and S-phase progression, respectively, and cyclins A and B (with Cdc2) are important for entry of the cells into the M phase (Schwartz and Shah, 2005). The activity of the cyclin/CDK complex can be inhibited by Cyclin-Dependent Kinase inhibitors (CDKi) such as p21^WAF1/Cip1^ and p27^kip1^ (Besson et al., 2008).

Over the past two decades, ion channels have been identified as important contributors to cell proliferation of normal and cancerous cells (for review see Wonderlin and Strobl, 1996; Kunzelmann, 2005). Otherwise, K^+^ channels are thought to be important for setting the membrane potential and the driving force for Ca^2+^ influx and both mechanisms are considered crucial for volume regulation of growing cells (Nilius and Wohlrab, 1992; Kunzelmann, 2005; Lang et al., 2007; Ouadid-Ahidouch and Ahidouch, 2008). Moreover, it is increasingly recognized that K^+^ channels control cell proliferation through a novel signaling mechanisms triggered and modulated independently from ion fluxes (Hegle et al., 2006; Kaczmarek, 2006; Glassmeier et al., 2012).

Although the K^+^ channels contribute to regulation of different cell cycle checkpoints in cancer cells, no direct evidence about the mechanism at issue has been provided concerning how they control the cell cycle machinery, particularly in regard to CDKi and/or cyclin expression (Evan and Vousden, 2001). This review focuses on the regulation of proliferation by K^+^ channel-mediated signals and discusses how these signals may influence the cell cycle progression in tumor cells.

## K^+^ channels and cell proliferation

K^+^ channels belong to a large and heterogeneous group of ion channels. The first K^+^ currents were described by Hodgkin and Huxley in the early 50s during their work on the identification of ionic species responsible for the propagation of the action potential in the squid giant axon. Today, several types of K^+^ channels have been identified by molecular cloning. They are responsible for the passive diffusion of K^+^ ion across the plasma membrane and are detectable in both excitable and non-excitable cells. K^+^ channels play a major role in several physiological functions such as synaptic transmission, muscle contraction, release of hormones, such as insulin (for review see Sandhiya and Dkhar, 2009), or in proliferation, apoptosis, migration, invasion, and angiogenesis (Pardo, 2004; Cherubini et al., 2005; Ouadid-Ahidouch and Ahidouch, 2008; Schwab et al., 2008; Becchetti, 2011; Cuddapah and Sontheimer, 2011; Jehle et al., 2011; Girault et al., 2012). The physiological importance of K^+^ channels is undoubtedly underscored considering the increasing number of genetic diseases associated with them. Indeed, Kv gene mutations trigger episodic ataxia, long QT syndrome, pancreatic diseases, and epilepsy (Ashcroft and Gribble, 2000; Goonetilleke and Quayle, 2012).

The early studies that have linked K^+^ channels to the proliferative processes were carried on T lymphocytes (DeCoursey et al., 1984; Matteson and Deutsch, 1984). In cancer, the first works were performed on melanoma cells, neuroblastoma, breast, lung, and bladder cancer cells (Rouzaire-Dubois and Dubois, 1990; Lepple-Wienhues et al., 1996; Wonderlin and Strobl, 1996; Ouadid-Ahidouch and Ahidouch, 2008). Since then, a wide variety of K^+^ channels has been shown to be implicated in the regulation of proliferation of myeloid leukemia, prostate, colon, neck, and head cancer, lymphoma and hepatocarcinoma cells (Zhou et al., 2003; Conti, 2004; Pardo, 2004; Wang, 2004; Kunzelmann, 2005; Wang et al., 2007a; Arcangeli et al., 2009; Jehle et al., 2011; Lehen'kyi et al., 2011). These data are based on the fact that, *in vitro*, genetic or pharmacological inhibition of K^+^ channels inhibits the growth of cancer cells. Moreover, K^+^ channel openers can also be responsible for an increase in proliferation rate. Indeed, minoxidil or cromakalim increased activity of K_ATP_ channels inducing an increase in DNA synthesis (Malhi et al., 2000). KCa3.1 channels activator, 1-EBIO, increased prostate cancer cell proliferation (Parihar et al., 2003), but diminished the proliferative effect on keratinocytes (Koegel et al., 2003).

Several hypotheses argue in favor of a mechanism of K^+^ influence on the cell cycle involving Ca^2+^ signaling, membrane potential, and cell volume (Nilius and Wohlrab, 1992; Lepple-Wienhues et al., 1996; Wonderlin and Strobl, 1996; Kunzelmann, 2005; Higashimori and Sontheimer, 2007; Zhanping et al., 2007; Ouadid-Ahidouch and Ahidouch, 2008; Blackiston et al., 2009; Becchetti, 2011).

One of the most accredited models is based on the involvement of K^+^ channels in the regulation of membrane potential, which drives cancer cells into certain phases of the cell cycle. In cancer cells, it has been shown that cells in the early G_1_ phase are depolarized; however, they are hyperpolarized during the progression through G_1_ and into the S phase (Wang et al., 1998). Inhibition of K^+^ channels or blocking cells in G1 phase by serum starvation is accompanied by membrane depolarization (Nilius and Wohlrab, 1992; Wonderlin et al., 1995; Wang et al., 1998; Ouadid-Ahidouch et al., 2001). Similarly, membrane depolarization caused by an increase in the concentration of extracellular K^+^ mimics the effects of K^+^ channel blockers (Freedman et al., 1992).

Many K^+^ channels have been implicated in the regulation of the membrane potential, (for review see: Wonderlin and Strobl, 1996; Pardo, 2004; Kunzelmann, 2005; Ouadid-Ahidouch and Ahidouch, 2008; Becchetti, 2011). In some cases, the membrane potential of cycling cells is regulated by the ratio of different K^+^ channel isoforms. For example, in neuroblastoma, the membrane potential can be controlled by Kv11.1 and blocking this channel inhibits mitosis (Arcangeli et al., 1995; Crociani et al., 2003; Becchetti, 2011). In these cells, the membrane potential tends to depolarize during the S phase. The effect is caused by oscillation of the expression balance of the full-length Kv11.1a isoform and the N-deleted Kv11.1b (Crociani et al., 2003). Indeed, the truncated Kv11.1b form is up-regulated during the S phase, while the full-length Kv11.1a protein increases its expression on the plasma membrane during the G1 phase (Arcangeli et al., 1995). The increase of the ratio between Kv11.1b and Kv11.1 leads to the depolarization of the membrane potential occurring during S phase progression (Arcangeli et al., 1995).

Ca^2+^ signaling appears to be required for progression through G1, the G1/S transition, and G2/M in several cell types (Kahl and Means, 2003; Roderick and Cook, 2008; Ding et al., 2010). The relationship between K^+^ channels, membrane potential, and Ca^2+^ influx has been first reported in T cells by Cahalan's group. Indeed, the authors proposed that the membrane hyperpolarization generated by the activation of K^+^ channels increases the driving force for Ca^2+^ influx (Hess et al., 1993; Lewis and Cahalan, 1995), which in turn, activates Ca^2+^-dependent transcriptional factors leading to expression of cell cycle regulatory proteins, such as cyclins, CDKs, and the inhibition of CDKi expression (Nilius and Wohlrab, 1992; Yao and Kwan, 1999; Ouadid-Ahidouch et al., 2004; Zhanping et al., 2007; Borowiec et al., 2011). The inhibitors of voltage-gated K^+^ channels block the Ca^2+^ influx and the cell proliferation (Yao and Kwan, 1999; Ouadid-Ahidouch et al., 2004; Zhanping et al., 2007).

In agreement with these general considerations, in prostate LNCaP cells, a member of Transient Receptor Potential (TRP) channel family, TRPV6 was identified as a major provider of passive calcium influx in response to hyperpolarization associated with activation of the intermediate-conductance Ca^2+^-activated K^+^ channel (KCa3.1) (Lallet-Daher et al., 2009). The same team showed that calcium entry through TRPV6 channel in these cells induced a subsequent downstream activation of Nuclear Factor Activated T cell (NFAT) leading to cell proliferation (Lehen'kyi et al., 2007). In breast cancer cells, it was demonstrated that Eag1 (Kv10.1) K^+^ channel function is required for controlling the Ca^2+^ entry trough Orai1 channels (Hammadi et al., 2012).

K^+^ channels also control cellular proliferation by affecting cell volume (Rouzaire-Dubois et al., 2000). Alterations of cell volume require participation of ion transport across cell membrane and accordingly, Cl^−^ and K^+^ channels (Lang et al., 2007). Recently, aquaporin channels have emerged as regulators of cell cycle progression (Di Giusto et al., 2012). Cell volume regulation may favor maintenance of appropriate levels of critical cell cycle regulatory proteins that are necessary for controlling cell cycle progression through G1 to S or G2/M phases (Tao et al., 2008; Huang et al., 2012).

In addition to their ability to permit ions to cross the membrane, ion channels can also have non-conducting functions that enable them to interact with cell signaling pathways to directly regulate biochemical events (Kaczmarek, 2006). It has been demonstrated that expression of a mutant form of Eag1, that cannot conduct current, promotes proliferation of NIH3T3 fibroblasts and C2C12 myoblasts cells (Hegle et al., 2006). In contrast to the wild type Eag1, the no-conducting Eag1 is independent of serum and is unaffected by changes in extracellular Ca^2+^. Moreover, proliferation induced by Eag1 is unaffected by changes in extracellular Ca^2+^, suggesting that increased Ca^2+^ influx is not an essential downstream component of Eag1-induced signaling. The same authors reported that Eag1 is able to regulate cell proliferation in fibroblasts via activation of the p38 Mitogen-Activated Protein (MAP) kinase pathway but not extracellular signal-regulated kinase 1/2 (ERK1/2). Ectopic expression of pore-dead mutant Eag1, in CHO cells, promotes cell growth *in vitro* and *in vivo* (xenografts) (Downie et al., 2008). Recently, the ion-conducting function of HERG1 has also proved to be important for cell growth of human small cell lung cancer cells (Glassmeier et al., 2012). Indeed, the knockdown of HERG1 inhibits cell proliferation, while its pharmacological inhibition by E4031 fails to affect cell proliferation (Glassmeier et al., 2012). Additionally, the expression of non-conducting mutant KCa3.1 induced HEK293 cells proliferation not by enhancing Ca^2+^ entry but via a direct interaction with ERK1/2 and c-jun N-terminal kinase (JNK) signaling (Millership et al., 2011). Increasing number of reports show that certain K^+^ channels interact with signaling molecules directly to regulate cellular signaling. Indeed, the N- and C-terminal domains of hEag1 interacts with calmodulin (Schönherr et al., 2000; Ziechner et al., 2006; Gonçalves and Stühmer, 2010), cortactin (Herrmann et al., 2012); KCa3.1 channels interact with ERK1/2 (Millership et al., 2011), and HERG1 channels with the adaptor protein 14-3-3 (Kagan et al., 2002), Src tyrosine kinase (Cayabyab and Schlichter, 2002), and the TNF-α receptor (Wang et al., 2002). In addition, HERG channel proteins have been shown to interact with integrins, thereby regulating cell survival, adhesion and migration (Arcangeli and Becchetti, 2006; Arcangeli, 2011).

The mechanisms that allow K^+^ channels to regulate cell growth in cancer cell lines appear to be different from the ones in normal cells. It has been reported that cancer cells express several K^+^ channel isoforms that may be physiologically different as compared to a wild type channel. For example, proliferation of neuroblastoma cells is regulated by an oscillation balance of expression of the full-length HERG 1a (Kv11.1a) isoform and the N-deleted HERG 1b (Kv11.1b) (Crociani et al., 2003). It should be also noted that the effects of K^+^ channels on cell proliferation can involve their trafficking to the micro-domains within the cell.

It follows from the foregoing that the K^+^ channels influence cell proliferation through as many mechanisms as the number of their families and correspondent isoforms. That is undoubtedly dependent on intrinsic features of each cell type and the isoforms expressed.

## K^+^ channel activity during the cell cycle

A direct link between channel activity and particular stages of the cell cycle has been reported. For example, in HeLa cells, the K^+^ current-density increases during M and G1 phases (Takahashi et al., 1993), in unfertilized mouse oocytes, a large-conductance, voltage-activated K^+^ channel (BKCa, 240 pS), is active throughout M and G1 phases, and switches off during the G1-to-S transition (Day et al., 1993). In Xenopus oocytes, the expressed rat Eag (rEag1) rEag1 displays decrease in current-density in meiotic phase induced by progesterone or by Mitosis promoting factor (Brüggemann et al., 1997). Furthermore, the partial synchronization of cells in G1 or M phases greatly increases the current blockade by intracellular Na^+^ and Cs^+^ (Pardo et al., 1998). In cancer cells, the K^+^ channels activity has been also found to be cell cycle-dependant. For example, the activity of Eag1 is at high rate in G1 phase and decreases when cells enter S phase (Ouadid-Ahidouch et al., 2004), and in neuroblastoma cells, HERG current activity has been shown to be cell cycle-dependent (Arcangeli et al., 1995).

The mechanisms linking the activity of each of these channels to the cell cycle appear to be different and include regulation by cytoskeletal elements (Camacho et al., 2000), the activation of cyclin-dependent kinase 1 (CDK1) cyclin B (Brüggemann et al., 1997), a cytoplasmic cell cycle that can run independently of the nuclear cell cycle (Day et al., 1998), or channel trafficking.

Studies on K^+^ channels trafficking are increasingly emergent. It has been suggested that Eag1 turns over rapidly (8–12 h) at the cell surface (Weber et al., 2006). This process involves surface expression followed by constitutive internalization and degradation in lysosomes (Kohl et al., 2011). Indeed, the Eag1 trafficking has been reported to be regulated by several proteins including cortactin, rabaptin-5 and epsin. Depletion of cortactin decreases the number of functional Eag1 channels without altering their open probability or conductance (Herrmann et al., 2012). These authors suggest that cortactin connects Eag1 to the cytoskeleton. Moreover, knockdown of rabaptin-5 reduces recycling rates of Eag1 and leads to a reduction of Eag1 current-density (Ninkovic et al., 2012). Eag1 also interacts with epsin that modulates its gating in rat brain (Piros et al., 1999). In view of these studies, the expression of K^+^ channels during the cell could likely due to their trafficking, although the involvement of this particular pathway in cell proliferation is still not proven.

## Molecular mechanisms underneath cell cycle regulation by K^+^ channels (see Table 1 and Figure 1)

Aberrantly expressed cell cycle-related cyclins are highly associated with several cancer types including breast cancer (for review Williams and Stoeber, 1996). As such, cyclin D1 is overexpressed in ~50% of breast cancers (Alle et al., 1998). Cyclin E is overexpressed in 30% of breast tumors (Wang and Shao, 2006), and the elevation of cyclin E is correlated with low levels of p27^Kip1^. A decrease in the expression of p27^Kip1^ is strongly detected in breast cancer tissues (for review, Alkarain et al., 2004). Further genetic abnormalities also affect other CDKi, such as p16^INK4a^, p15^INK4b^, and p57^Kip2^ (for review, Williams and Stoeber, 1996). These observations led to consider the cell cycle regulators as potential targets for selective inhibition in the treatment of cancer (Stone et al., 2012). The studies conducted so far show that the modulation of these key proteins of the cell cycle correlates with tumor development. Thus, it is clear that mitogenic factors capable of modulating their expression positively can intensify the process of carcinogenesis.

**Table 1:** **K^+^ channels and cell cycle protein actors in cancers**.

**Cell line type**	**K^+^ channel family**	**Cell cycle phase**	**Main actors**	**References**
MCF-7 (Breast cell line)	hEag1 (Kv10.1; KCNH1)	G1 and G1/S	Cyclin D1	Borowiec et al., 2011
			Cyclin E	
			P-Rb	
			No/CDK4	
			No/CDK2	
			No/p21^waf1/cip1^	
			No/p27^kip1^	
LoVo (Human colon cell line)	KCa3.1 (IK1; IKCa1; KCNN4)	G2/M	p-Cdc2	Lai et al., 2011
549 (Human lung cell line)	Kv1.3 (KCNA3)	G1	p21^Waf1/Cip1^	Jang et al., 2011
			CDK4	
			Cyclin D3	
U87 and U251 (Human glioma cell lines)	Kir6.2 (KCNJ11) ATP-sensitive	G1	p-ERK	Huang et al., 2009
LNCaP	KCa3.1 (IK1; IKCa1; KCNN4)	Prolif (G1/S)	p2^Cip1^	Lallet-Daher et al., 2009
PC-3			No/p27^Kip1^	
DU-145 (Prostate cancer cell lines)				
MB (Medulloblastoma) CNS tumor	EAG2 (Kv10.2; KCNH5)	G2	Cyclin B1 p38 MAPK	Huang et al., 2012
HL-60 (leukemia cells)	HERG1 (Kv11.1)	G1	β -catenin, cyclin-D1	Zheng et al., 2011
			c-myc	

**Figure 1:**
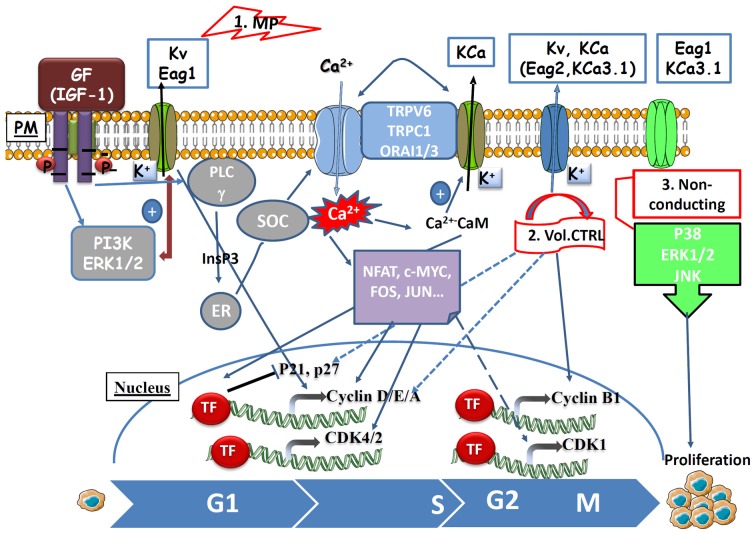
**Schematic illustration of the role of K^+^ channels during cell cycle progression and proliferation.** (1) Membrane potential model: during progression into G1 phase, the membrane potential becomes hyperpolarized relative to the normal resting potential. The hyperpolarization relates to Kv channels activation (for example Kv1.3, Eag1) in early G1 and to Ca^2+−^activated K^+^ channels (for example KCa3.1) activation in late G1 and/or G1/S transition. Multiple growth factors in serum have been well-described to play an important role in initiating G1 progression to the S phase of the cell cycle, in which Ca^2+^ influx is a major determinant in serum-induced DNA synthesis. Growth factors (for example IGF-1) binding to the receptor tyrosine kinases [RTKs; for example, Insulin growth factor receptor (IGF-1R)] can activate effectors [Extracellular signal-Regulated Kinase 1/2 (ERK1/2), phosphatidylinositol 3-kinase (PI3K)] that increase the expression and the activity of K^+^ channels inducing a hyperpolarization of the membrane potential (MP). Moreover, RTKs also activate phospholipase C (PLCγ) to promote the generation of inositol-1,4,5-trisphosphate (InsP3) and the release of Ca^2+^ from the endoplasmic reticulum (ER) into the cytosol. Ca^2+^ enters across plasma membrane by store-operated capacitive Ca^2+^ entry (SOCE) through the ORAI1 (Hammadi et al., 2012) or ORAI3 (Faouzi et al., 2011) channels, through TRP Ca^2+^ channels (for example TRPC1, El Hiani et al., 2009), or via the constitutively active TRPV6. The Ca^2+^ entry, in turn, regulates the activity and/or the expression of Ca^2+^-activated K^+^ channels (for example KCa3.1), which maintains the hyperpolarization promoting a significant Ca^2+^-entry. The increase in [Ca^2+^]i triggers the activation of Ca^2+^-dependent signaling enzymes that may act by regulating the expression or activity of the transcription factors (TF), such as FOS, JUN, NFAT, C-MYC leading to the expression of cyclins and CDKs and the inhibition of the CDK inhibitor proteins (p27^KIP1^ and p21^waf1/cip1^). (2) Volume control: K^+^ channels in association with chloride channels regulate cell cycle progression by controlling the cell volume. For example, Eag2 channels control M phase by regulating the expression of cyclin B1 through the p38 MAP Kinase pathway. (3) Non-conducting roles: K^+^ channels may also promote cell proliferation independently of their ion permeation function. For example: Eag1 and KCa3.1 induce cell proliferation by (direct or indirect) interaction with MAP kinase signaling pathways (p38 for Eag1, ERK1/2, and JNK for KCa3.1). MP: membrane potential, (vol. CTRL): volume control, (PM): plasma membrane.

Despite K^+^ channel blockers modulate several proliferative signaling pathways evidence for a direct mechanistic link between K^+^ channels and cell cycle proteins remains sparse to date. Two cell-cycle blocks are often observed when K^+^ channels are inhibited: at G1 (early G1, late G1, G1/S transition) and at G2/M block with or without changes in the proportion of the cells entering S-phase. The G1 block is associated with a reduction of the expression of cyclins (D and E), CDKs (4 and 2), pRb activation, and overexpression of CDKi (p21^Waf1/Cip1^ and p27^kip1^), while the block in G2/M is associated with a reduction of cyclin B1 expression or changes in Cdc2 (CDK1) phosphorylation.

The first studies used K^+^ channels pharmacological inhibitors such as TetraEthyl Ammonium (TEA) and 4-Amino-Pyridine (4-AP) to demonstrate that inhibition of the two K^+^ channels (Kv1.3 and Kv1.5) resulted in cell cycle arrest at G1 in rat oligodendrocyte precursors (Ghiani et al., 1999; Chittajallu et al., 2002). The authors proposed that changes in the membrane potential (depolarization) could activate a signaling pathway involving the p27^kip1^ and p21^Waf1/Cip1^ proteins expression. Similar results were obtained when the membrane potential was depolarized by increasing extracellular K^+^ (Renaudo et al., 2004).

In small cell lung cancer (SCLC, NCI-H209, and NCI-H146) and leukemic (Jurkat) cell lines, inhibition of K^+^ channels has also been implicated in upregulation of the p27^kip1^ protein and a reduction in cyclin. A expression resulting in cell arrest in G1/S phase transition (Renaudo et al., 2004). Athough the mechanisms, by which K^+^ channels regulate cell cycle in these cells remain undetermined, the authors suggested involvement of cytoskeleton rearrangements due to cell volume changes.

A detailed mechanism underlying the role of Eag1 in the cell cycle was studied in MCF-7 breast cancer cells. In this work, mitogenic stimulation [using serum or Insulin Growth Factor 1, (IGF-1)] up-regulates Eag1 expression and activity (Borowiec et al., 2007). IGF-1 triggers cell cycle progression by increasing expression of cyclin D1, cyclin E, CDK4, CDK2, and phosphorylation of pRb (Dufourny et al., 1997; Borowiec et al., 2011). Inhibition of Eag1 by astemizole or by siRNA induced a decrease in cyclin D1 expression along with a strong decrease of pRb phosphorylation and an arrest of the cells in G1 phase of cell cycle. The effect of Eag1 inhibition was accompanied by a decrease of cyclin E expression, the key regulator of the G1/S transition necessary for cell entry into S phase (Skelding et al., 2011). However, inhibition of Eag1 failed to modify the p21^Waf1/Cip1^, p27^Kip1^, and CDK4/2 expression.

Interestingly, when Eag1 is inhibited, the cyclin D1 level is below its level in serum-deprived cells, the condition known to synchronize cells in G1 phase (Borowiec et al., 2011). It was thus suggested that Eag1 control of the cell cycle could be upstream of the G1 phase synchronization by serum deprivation. Indeed, the cell cycle arrest site under Eag1 inhibition is both upstream of that obtained by serum-deprivation, and downstream of that induced by lovastatin, which synchronizes the cells in the early G1 phase (Borowiec et al., 2011). Altogether, these results demonstrate that Eag1 activation is positioned upstream of the upregulation of both cyclins D1 and E expression.

It has been reported recently that the isoform 2 of Eag (Eag2) is essential for mitotic entry of medulloblastoma (MB) cells (Huang et al., 2012). Indeed, the authors observed a correlation between temporal Eag2 translocation to plasma membrane and mitotic entry from the late G2 phase concurrent with an increase of outward K^+^ current during mitosis. Moreover, Eag2 knockdown accumulates cells in G2/M in association with a strong decrease of cyclin B1 (essential for G2/M transition), but fails to affect the expression of cyclin A, cyclin D1, cyclin E, and CDK1 (Huang et al., 2012). The G2/M arrest is due to an alteration in cell volume control that is linked to hyperactivation of p38 MAPK pathway without any effect on p53 expression. It has been then hypothesized that Eag2 regulates cell volume during the MB cell cycle progression (Habela and Sontheimer, 2007; Boucrot and Kirchhausen, 2008).

Ca^2+^-activated K^+^ channels (KCa) also regulate cell cycle progression. KCa3.1 is one of the KCa channels that regulate proliferation and migration of cancer cells (Ouadid-Ahidouch et al., 2004; Faouzi et al., 2010; Catacuzzeno et al., 2012). KCa3.1 controls G1 (mainly late G1) phase, G1/S transition, and G2/M phase. In breast, prostate and endometrial cancers, KCa3.1 regulates G1 and G1/S transition (Ouadid-Ahidouch et al., 2004; Wang et al., 2007b; Lallet-Daher et al., 2009). Pharmacological or genetic blockade of KCa3.1 increases p21^Waf1/Cip1^ expression and decreases the expression of cyclin E. According to the “potential membrane model,” activation of K^+^ channels amplifies the Ca^2+^ signals by hyperpolarizing the membrane potential, thus increasing driving force for Ca^2+^ influx, which, in turn, activates Ca^2+^-dependent transcriptional factors leading to expression of G1 cyclins and CDK proteins (Roderick and Cook, 2008). Consistent with this notion, it has been shown that TRPV6 may be the major provider of passive Ca^2+^ influx in response to the hyperpolarization associated with KCa3.1 channels activation in prostate cancer cell line (Lallet-Daher et al., 2009). In breast cancer cells, it has been demonstrated that Eag1, by regulating membrane potential, controls Ca^2+^ entry through Orai1 channels (Hammadi et al., 2012).

KCa3.1 channels also control the cell volume and regulate G1/S transition and G2/M phases (Tao et al., 2008; Lai et al., 2011). In mouse mesenchymal stem cells, KCa3.1 channels associated with Cl^−^ -volume channels regulate cell cycle progression by modulating cyclin D1 and cyclin E expression (Tao et al., 2008). However, in human colon cancer cells (LoVo), their inhibition accumulates cells in G2/M phase and increases the phosphorylation of Cdc2 (Lai et al., 2011).

In summary, several K^+^ channels have been implicated in proliferation of various types of cancers cells including Kv (Menéndez et al., 2010; Asher et al., 2011; Jang et al., 2011; Jeon et al., 2012), KCa (Jäger et al., 2004; Faouzi et al., 2010; Oeggerli et al., 2012), Kir (Huang et al., 2009), K2P (Bayliss and Barrett, 2008; Innamaa et al., 2013). Higher expression or activity of these channels in cancer cells appears to correlates with deregulation of cell cycle protein expression and/or function leading to enhanced cell proliferation.

## Conclusions

In summary, K^+^ channels are often overexpressed in tumor cells and regulate proliferation. They are needed to control specific checkpoints in the cell cycle progression (early G1, the G1/S and G2/M transitions). Accordingly, several K^+^ channels exhibit a cell-cycle-dependent expression and activity. In addition, cell cycle progression is often accompanied by oscillations of Cl^−^ channel expression and Ca^2+^ signals that regulate cell proliferation. Recent evidence shows the involvement of aquaporin in cell proliferation. The links between K^+^ channels and cell-cycle machinery (cyclins, CDKs, and CDKi) start to emerge (Figure 1). However, we are still far from full understanding the complex mechanistic link between channel expression/activity and signaling in proliferating cells. The detailed understanding of the role of K^+^ channels and their connection with Ca^2+^ signals in regulation of cell cycle proteins and/or transcription factors will offer significant opportunities to develop more specific tools for cancer therapy.

### Conflict of interest statement

The authors declare that the research was conducted in the absence of any commercial or financial relationships that could be construed as a potential conflict of interest.
